# Antioxidant and α-amylase Inhibitory Activities and Phytocompounds of *Clausena indica* Fruits

**DOI:** 10.3390/medicines7030010

**Published:** 2020-02-28

**Authors:** La Hoang Anh, Tran Dang Xuan, Nguyen Thi Dieu Thuy, Nguyen Van Quan, Le Thu Trang

**Affiliations:** Department of Development Technology, Graduate School for International Development and Cooperation, Hiroshima University, Hiroshima 739-8529, Japan; hoanganh6920@gmail.com (L.H.A.); dieuthuykttb@gmail.com (N.T.D.T.); nguyenquan26@gmail.com (N.V.Q.); trangle9872@gmail.com (L.T.T.)

**Keywords:** *Clausena indica*, fruits, antioxidants, α-amylase inhibitor, antidiabetes, gas chromatography-mass spectrometry

## Abstract

**Background:** Clausena indica fruit is commonly used for food ingredients and traditional medicines in tropical countries, however, information about its biological activities and chemical profiles has been inadequately reported. **Methods:** In this study, a bio-guided fractionation of antioxidants and α-amylase inhibitors from hexane (MH) and ethyl acetate (ME) extracts of *C. indica* fruit (pericarp and seed) was carried out. Eleven fractions from MH (D1–D11) and 17 fractions from ME (T1–T17) were obtained from column chromatography over silica gel, which were then examined for anti-radical capacity by 2,2-diphenyl-1-picrylhydrazyl (DPPH) and 2,2′-azinobis-(3-ethylbenzothiazoline-6-sulfonic acid) (ABTS) assays, and pancreatic α-amylase inhibition, a key enzyme linked to type 2 diabetes. **Results:** Of isolated fractions, the fraction T4 revealed the most potent anti-DPPH activity (IC50 = 0.13 mg/mL), whereas T2 exhibits the strongest ABTS cation scavenging ability (IC50 = 0.31 mg/mL). In the enzymatic assay, the fractions D3 and T4 significantly inhibit the α-amylase reaction with IC50 values of 0.34 and 0.86 mg/mL, respectively. Remarkably, α-amylase suppression of T4 is close to acarbose and over four times stronger than palmitic acid, which are the well-known α-amylase inhibitors (IC50 = 0.07 and 1.52 mg/mL, respectively). The active constituents from fractions were identified by gas chromatography-mass spectrometry (GC-MS). The results show that the fraction D3 contains five major compounds, which are grouped in five classes consisting of fatty acids, phenols, benzodioxoles, alcohols, and sesquiterpenes. Among them, palmitic acid is the most dominant compound (32.64%), followed by 2R-acetoxymethyl-1,3,3-trimethyl-4t-(3-methyl-2-buten-1-yl)-1t-cyclohexanol (16.69%). Whilst, six major compounds belonging to fatty acid and coumarin classes are identified in the fraction T4. The most abundant compound in T4 is dentatin (47.32%), followed by palmitic acid (15.11%). **Conclusions:** This is the first finding that *C. indica* fruit can be a promising source for the development of natural antioxidant and antidiabetic agents. Additionally, the outcome reveals that dentatin, a known natural antineoplastic agent, can be feasibly exploited from *C. indica* fruit.

## 1. Introduction

*Clausena indica* is an evergreen shrub belonging to the citrus Rutaceae family, which is popularly grown in South China, and South and Southeast Asia. The plant parts of *C. indica* have been widely used for multiple purposes including foodstuffs and folk medicines. For example, the plant’s leaves and roots can be applied as a traditional medicine to treat a variety of health problems, such as colds, flu, headaches, colic and rheumatism, joint dislocation, and bone fractures. The essential oil from *C. indica* leaves exhibits antimicrobial and antibacterial activities. In addition, fruits of this plant are widely used in cooking as natural spices, which can improve the aromatic flavor of food [1,2]. A wide range of phytochemical components can be isolated from the *Clausena* genus, of which coumarins, carbazole alkaloid, and sesquiterpenes are the most abundant compounds, which might have potential for anticancer activity [3,4,5,6]. In the previous study of Quan et al. [7], *C. indica* roots were announced to have multiple biological activities comprising of antioxidant, anti-α-amylase and anti-tyrosinase. In addition, three pure anti-carcinogenic compounds including dentatin, nordentatin, and clausine K were isolated and purified from *C. indica* roots [7]. Therefore, in this study, the beneficial constituents of *C. indica* fruits in terms of antioxidants and anti-diabetes are continuously identified.

In all organisms, especially humans, oxidation is a fundamental metabolic process to produce energy. However, redox reactions generate invariably reactive oxygen species (ROS). Noticeably, ROS are well known undesirable products leading to the development of oxidative stress, which involves in many human diseases such as diabetes, skin pigmentation, obesity, and cancer [8]. Furthermore, type 2 diabetes is considered as one of the most chronic diseases that is regulated by oxidative stress via molecular mechanisms [9]. Among diabetes cases, type 2 diabetes is more common and appears in different ages [10]. In type 2 diabetes, the body is resistant to insulin resulting in an increase in sugar level in blood as a typical symptom. The level of sugar in the blood is determined by the activity of digestive carbohydrate-hydrolyzing enzymes, notably α-amylase, which acts on starch. The long-chain carbohydrates of starch, amylose, and amylopectin are broken down into glucose by the catalysis of α-amylase in the digestive process. As a result, glucose is released into the bloodstream [11]. For that reason, inhibition of α-amylase activity can reduce sugar levels in the blood, which can be a promising solution for diabetes avoidance. Furthermore, the gathering of antioxidants and suppression of α-amylase is a prospective approach for the treatment of type 2 diabetes.

Based on the above-mentioned reasons, this research was conducted with the aim of establishing bio-guided fractionation of bioactive constituents from *C. indica* fruits and to investigate their potential for antioxidants and α-amylase suppression via in vitro assays. The findings of this study could provide relevant information for a comprehensive view of *C. indica* species values in particular, and the *Clausena* genus in general.

## 2. Materials and Methods

### 2.1. Materials

The fruits of *C. indica* were purchased from a local market in Thai Nguyen City, Thai Nguyen Province, Viet Nam in September 2018. The fruit sample was authenticated by Phung Thi Tuyen, Vietnam National Forestry University. The identification of the species was mainly based on Vietnam Plant Data Center (http://www.botanyvn.com) and Plants Database Missouri Botanical Garden, United States (TROPICOS-http://www.tropicos.org) and The Plant List (http://www.theplantlist.org). The voucher specimen number MMF-J2018 was preserved at the Laboratory of Plant Physiology and Biochemistry, IDEC, Hiroshima University, Japan.

### 2.2. Sample Preparation and Extraction

The pre-dried *C*. *indica* fruits (pericarp and seed) were washed with water several times before soaking in 0.5% sodium hypochlorite (NaClO) to remove impurities. After that, the samples were cleaned again with distilled water before being put in an oven at 40 °C for 5 days. Subsequently, the dried fruits were ground into a soft powder. In room temperature conditions, five liters of methanol was used 3 times repeatedly to extract the ground fruits (1.6 kg). After filtration, the solvent was evaporated at 50 °C by an evaporator to retrieve the methanol crude extract (162.6 g). Two hundred milliliters of distilled water was mixed with the obtained crude extract for the next step of the liquid–liquid phase extraction procedure. After that, two hundred milliliters of hexane and ethyl acetate, respectively, were also applied 3 times repeatedly to retrieve extracts. Lastly, the crude extracts were filtered and evaporated at 50 °C to attain the dried crude extracts.

### 2.3. Isolation of Bioactive Compounds

**Figure 1:**
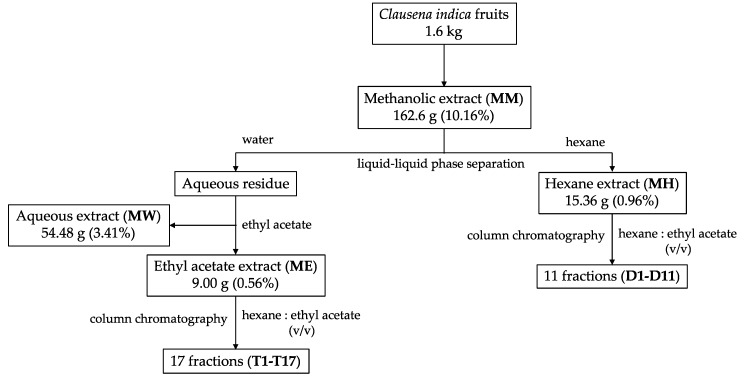
Extraction and isolation procedure of bioactive constituents from *C. indica* fruit.

The screening for the biological activities of methanol (MM), hexane (MH), ethyl acetate (ME) and water (MW) extracts were performed. Resultantly, MH and ME appeared to be the most active extracts and they were further fractionated by applying the column chromatography method. Fifty grams of silica gel (70–230 mesh) was adsorbed into a portion of MH or ME to get a mixed solution, which was loaded into the column afterward. The column was then eluted with a gradual gradient of the initial mobile phase by adding a mixture of n-hexane and ethyl acetate, followed by a mixture of ethyl acetate and methanol. Briefly, the liquid fraction was spotted on a TLC plate and run by a suitable solvent system, specifically, a mixture of n-hexane and ethyl acetate (v/v) in an oven at 100 °C for 2 min. Based on TLC results, similar fractions were bulked together. The separation of the bioactive fraction procedure from *C. indica* is precisely displayed in Figure 1.

### 2.4. Determination of Total Phenolic Content

The total phenolic content (TPC) of extracts from *C. indica* fruits was determined by the Folin–Ciocalteu method as described by Elzaawely et al. [12] with a slight alteration. Initially, twenty microliters of sample and 100 µL of 10% Folin–Ciocalteu’s reagent were blended, instantly followed by 80 µL of Na_2_CO_3_ 7.5% (w/v). The combination was then incubated at room temperature for 30 min. The outcome data was expressed as milligrams of gallic acid equivalent per one gram of sample dry weight (mg GAE/g DW).

### 2.5. Determination of Total Flavonoid Content

The total flavonoid content (TFC) was determined through the aluminum chloride colorimetric method described by Tuyen et al. [13]. The result was expressed as milligrams of rutin equivalent per one gram of sample dry weight (mg RE/ g DW). The mixture consisting of 50 µL of sample and 50 µL of AlCl_3_ (2%, w/v) was incubated for 20 min before being measured by a microplate reader (MR) at 430 nm.

### 2.6. Antioxidant Activity

Antioxidant ability of extracts and achieved fractions from *C. indica* fruits was determined by screening for anti-radicals via DPPH [14] and ABTS [15] assays as follows.

Based on the method presented by Elzaawely and Tawata [14], the in vitro DPPH assay was conducted via a slightly adjusted process. In detail, the reaction was performed by adding 80 µL of methanolic sample in microplate together with 40 µL of DPPH solution (0.5 mM) and 80 µL of 0.1M acetate buffer (pH = 5.5). The microplate was then overlaid to avoid light for 20 min at 25 °C. The inhibition on DPPH radical can be observed as the discoloration of the final solution and was evaluated as having a decreased absorbance at 517 nm.

Whereas, based on the research of Pellegrini et al. [15], the in vitro suppression against ABTS radical cation was conducted through a slightly alterative procedure. Briefly, the incubated reaction between 7 mM ABTS and 2.45 mM potassium persulfate (1:1, v/v) was protected from the effect of light for 16 h at room temperature to generate ABTS working solution. The collected solution was subsequently diluted with MeOH to obtain the absorbance of 0.70 ± 0.05 at 734 nm. The inhibitory effect of the sample (25 µL) on ABTS working solution (200 µL) was recorded after 20 min of incubation at 25 °C with the absence of light. The inhibition on ABTS radical cation can be determined by the decolorization of the final solution and reported in values as reduced absorbance at 734 nm.

Butylated hydroxytoluene (BHT) was selected as a positive control. On the contrary, methanol was the negative control. The formula for calculating radical scavenging percentage is displayed as follows:

radical scavenging activity (%) = [(1 − (S − B_s_)]/ (C − B_c_) × 100 (1)

where S is the absorbance of reaction with the sample or BHT, B_s_ is the absorbance of reaction without radical solution. C is the absorbance of the reaction with methanol, B_c_ is the absorbance of methanol without the radical solution. The required concentration for inhibiting 50% of radicals (IC_50_ value) was obtained via a linear equation acquired from the dose-response curve. Lower IC_50_ value means stronger inhibition.

### 2.7. Porcine Pancreatic α-Amylase Inhibition Assay

The inhibition on porcine pancreatic α-amylase (PPA) was based on the method demonstrated by Quan et al. [16]. The first step was to mix 20 µL of sample with 20 µL of PPA (20 units/mL in buffer). The mixture was then incubated for 10 min in the MR at 37 °C. Subsequently, thirty microliters of soluble starch (0.5%) was pipetted into the solution and continuously kept in MR at 37 °C for another 8 min. After stopping the reaction with 20 µL of HCl (1 M), one hundred microliters of iodine reagent (0.25 mM) was added. The inhibition of PPA resulted in the remaining of starch in the final solution, which reacted with iodine to turn the color to deep blue. Otherwise, light yellow means nonactive. The inhibition on PPA was determined by increased absorbance at 565 nm and based on the following formula:

% inhibition = (A − C)/(B − C) × 100(2)

where A is the absorbance of solution with the effect of sample, B is the absorbance without the reaction between enzyme and starch (replacing enzyme and starch by buffer), C is the absorbance of solution without samples. Acarbose and palmitic acid were used as a positive reference. Standard curves of percentage inhibition were plotted based on the mean values. The IC_50_ parameter was displayed similarly to the above-mentioned assays of DPPH and ABTS.

### 2.8. Identification of Phytochemical Component by Gas Chromatography-Mass Spectrometry (GC-MS)

The phytochemical constituents of MH and ME fractions were elucidated using GC-MS analysis. The applied GC-MS system (JMS-T100 GCV, JEOL Ltd., Tokyo, Japan) in the present study is equipped with an autosampler connected with a DB-5MS column (30 m × 0.25 mm I.D. × 0.25 µm film thickness) (Agilent Technologies, J&W Scientific Products, Folsom, CA, USA). Samples (1000 µg/mL) were prepared and placed in the autosampler. The samples were then automatically injected into the GC-MS system. The carrier gas helium was used at a split ratio of 5:1. The temperature condition of the GC oven was established as follows: the beginning temperature started at 50 °C and without holding, the temperature was rushed to 300 °C (10 °C /min) and maintained for 20 min. The applied temperatures for the injector and detector were 300 °C and 320 °C, respectively. The mass ranged from 29 to 800 amu for scanning. The analysis outcome was confirmed via the library of GC-MS Mass Center System version 2.65a.

### 2.9. Statistical Analysis

All bioassays were carried out in triplicate. The statistical analysis was performed by one-way ANOVA using the Minitab version 16.2.3. Regarding the significant differences, the means ± standard deviation (SD) were performed in various groups, applying Turkey’s test at *p* < 0.05.

## 3. Results

### 3.1. Extraction Yield and Total Phenolic and Flavonoid Contents of Extracts from C. indica Fruits

A total of 162.6 g (10.16%) of methanol extract (MM) was obtained from 1.6 kg of dried *C. indica* fruits. Hexane (MH), ethyl acetate (ME) and water (MW) extracts are subsequently collected with the amounts of 15.36 g (0.96%), 9.00 g (0.56%), and 54.48 g (3.41%), respectively.

The total phenolic (TPC) and flavonoid (TFC) contents of extracts from *C. indica* fruits are displayed in Table 1. The highest TPC level is found in MM (8.19 mg GAE/g DW), followed by MW, ME, MH with the amounts of 3.25, 0.66, and 0.20 mg GAE/g DW, respectively. Whilst, the highest TFC level is also found in ME (0.65 mg RE/g DW), the second-highest is MM (0.17 mg RE/g DW). MH and MW similarly show the lowest TFC levels (0.07 mg RE/g DW).

### 3.2. Antioxidant and Anti-α-Amylase Activities of C. indica Fruit Extracts

The antioxidant activity of *C. indica* fruit extracts is determined by screening for anti-radical ability via DPPH and ABTS assays. According to results described in Table 2, all extracts show antioxidant ability in both DPPH and ABTS assays. Remarkably, MM exhibits a strong antioxidant activity with the lowest IC_50_ values (0.12 and 0.26 mg/mL, respectively), followed by ME, MW and MH. However, only MH and ME display inhibition on α-amylase, while the other extracts show neither a negligible effect nor activity on the enzyme. Interestingly, MH (IC_50_ = 1.37 mg/mL) has a stronger anti-α-amylase ability than the positive control, palmitic acid (IC_50_ = 1.52 mg/mL). As a result, the extracts including MH and ME were selected for further fractionation and screening for biological activities.

### 3.3. Fraction Yields from C. indica Fruit Hexane and Ethyl Acetate Extracts

The MH and ME extracts were fractionated by column chromatography over silica gel. Based on the TLC method, similar fractions were mixed together. A total of 11 fractions from MH extract and 17 fractions from ME extract and their yields are listed in Table 3.

### 3.4. Antioxidant and Anti-α-Amylase Activities of Isolated Fractions from C. indica Fruit Extracts

Antioxidant properties of fractions from MH and ME extracts are displayed in Table 4. According to the results of the DPPH assay, the antioxidant activity is performed as IC_50_ values, which range from 0.13 mg/mL to 6.06 mg/mL. Particularly, the highest antioxidant activity is reported in T4 (IC_50_ = 0.13 mg/mL), while the lowest is determined in D1 (6.06 mg/mL). However, in the case of the ABTS assay, T2 shows the strongest ability (IC_50_ = 0.31 mg/mL), whereas D1 presents negligible inhibitory activity (IC_50_ = 12.44 mg/mL). The fractions from D3 to D8 show trivial inhibitory activities, thus, their IC_50_ values are not determined.

For α-amylase inhibitory activity, among screened fractions, T4 shows the highest inhibition (IC_50_ = 0.34 mg/mL), followed by D3 (IC_50_ = 0.86 mg/mL). The fractions from D5 to D10 show negligible effects on α-amylase, therefore their IC_50_ values cannot be evaluated.

### 3.5. Identification of Compounds by Gas Chromatography-Mass Spectrometry (GC-MS)

With the bioassay-guided separation by column chromatography, the antioxidant and enzyme inhibitory abilities of all extracts and fractions are determined. The results show that T4 possesses the strongest in both antioxidant and anti-α-amylase activities among fractions isolated from ME extract, while D3 shows the most promising suppression against α-amylase among the tested fractions from the MH extract. The identification of the main components using the GC-MS system are presented in Table 5.

In the fraction T4, six major compounds belonging to two classes are identified, which are fatty acids (palmitic, linoleic and oleic acids), and coumarin (seselin, braylin, and dentatin). In which, dentatin is the most dominant compound (47.32%), followed by palmitic acid (15.11%).

In the fraction D3, five compounds are identified. They can be grouped into five categories including fatty acids (palmitic acid), phenols (methoxyeugenol), benzodioxoles (myristicin), alcohols (13-tetradece-11-yn-1ol) and sesquiterpenes (S1). Among the detected compounds, palmitic acid is the most abundant (32.64%), followed by 2R-acetoxymethyl-1,3,3-trimethyl-4t-(3-methyl-2-buten-1-yl)-1t-cyclohexanol (16.69%).

## 4. Discussion

Numerous studies indicate that the genus *Clausena* exhibits various valuable bioactivities including anticancer [17], antibacterial [18,19,20], antioxidants [21,22,23], anti-HIV [24]. Besides, a wide range of substances belonging to diverse chemical classifications can be detected in *C. indica,* which have high potential for many biological activities [3,4,5,6]. However, bioactive components from *C. indica* have still received little attention from researchers, even though the plants are used widely as traditional medicines and foodstuffs. Via this research, for the first time, we attempt to discover comprehensively the antioxidants together with α-amylase inhibitory activity and relevant compounds in the fractions from *C. indica* fruit extracts.

In general, biological activities of a sample are determined by its compound content, in which functional groups of individual compounds have specific roles. For example, the existences of hydroxyl substituents and aromaticity in the structure of phenolic compounds are responsible for facilitating free radical scavenging [25]. In agreement, the total methanol extract from *C. indica* reveals the highest TPC and TFC together with the lowest IC_50_ values in both DPPH and ABTS assays. However, the water extract achieves second place for TPC levels, displaying an insignificant antioxidant activity in comparison to other extracts. It could be explained that phenolics are major compounds but not the only contributor to antioxidant activity. On the contrary, for enzymatic inhibitors, it is difficult to specify the important aspect of chemical structure. The α-amylase suppression might depend on various factors such as the hydroxyl position, methoxy groups, and lactone rings or the interaction between compounds [16]. As a result, different inhibitory activities of fractions from *C. indica* extracts are recorded in the present study. Among assayed fractions, T4 reveals the strongest antioxidant activity and α-amylase inhibition. Remarkably, inhibitory effect on α-amylase of T4 is close to acarbose, a popular anti-diabetes drug [26], and over four times stronger than palmitic acid, an acknowledged α-amylase inhibitor [27]. Moreover, D3 shows a negligible antioxidant capacity in DPPH and ABTS radical scavenging assays, however, the fraction presents a significant suppression against α-amylase. Consequently, the fractions T4 and D3 were selected to identify the major chemical constituents by applying the GC-MS technique. The results show that dentatin is the most dominant compound in T4. This anticancer substance can be found in a wide range of *Clausena* species [19,28,29,30]. On the other hand, dentatin was isolated and purified from *C. indica* roots in our previous study, in which, the compound was found to possess a negligible effect on antioxidants and no activity on anti-α-amylase [7]. Therefore, dentatin is not a major substance responsible for the potent biological activities of T4 but might have a role as a co-factor. The interaction between dentatin and other phytocompounds might result in strong antioxidant and α-amylase inhibitory activities of the fraction T4. Nevertheless, the mode of action, as well as the kinetic mechanism of these reactions should be further investigated. Beside dentatin, palmitic, linoleic, and oleic acids are abundant in T4. These fatty acids were reported as antioxidant substances, which might lead to the respective activity of T4 [31,32,33]. Interestingly, palmitic acid is found abundant in both fractions T4 and D3. Therefore, this compound might have a role as an important contributor, but not the only one, for a fraction’s anti-α-amylase ability. A wide range of inhibitory effects on α-amylase was recorded. The strongest α-amylase suppression of T4 might be explained by the synergistic effect of fatty acids including palmitic, linoleic, and oleic acids, which were announced as being involved in anti-diabetes activity [27,34,35]. Moreover, in previous studies, these fatty acids showed remarkable inhibition on α-glucosidase enzyme, which was significantly higher than acarbose [27,35]. Therefore, *C. indica* fruits could be predicted as having potential for anti-α-glucosidase activity, which is involved in the prevention of hyperglycemia [36]. If that is the case, a comprehensive solution for type 2 diabetes avoidance could be exploited from *C. indica* fruits.

T4 can be selected for the further isolation and purification of potentially bioactive compounds. Moreover, clinical investigations and in vivo approaches should be implemented to confirm pharmaceutical benefits of *C. indica* fruits against type 2 diabetes in particular and other chronic diseases caused by oxidative stresses in general.

## 5. Conclusions

Our study is the first investigation of antioxidant and anti-α-amylase capacities of *C. indica* fruits. Among fractions, T4 from ethyl acetate extract shows the highest suppression against α-amylase together with potent antioxidant activity. The identification of chemical constituents by the GC-MS system suggests that a known anti-cancer compound (dentatin) is rich in fraction T4. In addition, palmitic acid might contribute a preeminent role to α-amylase inhibitory ability of *C. indica* fruits. The findings suggest that fruits of *C. indica* are a promising source of antioxidants and α-amylase inhibitors, which are prospectively utilized for the prevention of type 2 diabetes. Further isolation and purification should be conducted to exploit and discover prospective active compounds from this plant.

## Figures and Tables

**Table 1:** Total phenolic and flavonoid contents of extracts from *C. indica* fruits.

Extracts	TPC (mg GAE/g DW)	TFC (mg RE/g DW)
**MM**	8.19 ± 0.09 ^d^	0.17 ± 0.00 ^b^
**MH**	0.20 ± 0.01 ^a^	0.07 ± 0.00 ^a^
**ME**	0.66 ± 0.00 ^b^	0.65 ± 0.02 ^c^
**MW**	3.25 ± 0.02 ^c^	0.07 ± 0.00 ^a^

The data express means ± standard deviation (SD). Different letters in a column indicate significant differences at *p* < 0.05. TPC, total phenolic content; TFC, total flavonoid content; GAE, gallic acid equivalent; RE, rutin equivalent; DW, dry weight; MM, total methanolic extract; MH, hexane extract; ME, ethyl acetate extract; MW, water extract.

**Table 2:** Antioxidant and anti-α-amylase activities of *C. indica* fruit extracts.

Samples	DPPH IC_50_ (mg/mL)	ABTS IC_50_ (mg/mL)	α-Amylase IC_50_ (mg/mL)
**MM**	0.12 ± 0.00 ^b^	0.26 ± 0.00 ^b^	*ne*
**MH**	1.45 ± 0.00 ^e^	1.49 ± 0.00 ^e^	1.37 ± 0.01 ^b^
**ME**	0.16 ± 0.00 ^c^	0.35 ± 0.00 ^c^	8.56 ± 0.24 ^a^
**MW**	0.17 ± 0.00 ^d^	0.43 ± 0.00 ^d^	*na*
**BHT**	0.02 ± 0.00 ^a^	0.06 ± 0.00 ^a^	-
**Acarbose**	-	-	0.07 ± 0.00 ^c^
**PA**	-	-	1.52 ± 0.03 ^b^

The data express means ± standard deviation (SD). Different letters in a column indicate significant differences at *p* < 0.05. MM, total methanolic extract; MH, hexane extract; ME, ethyl acetate extract; MW, water extract; BHT, butylated hydroxytoluence; PA, palmitic acid; “-“, not measured; *ne*, negligible effect; *na*, no activity.

**Table 3:** Column chromatography procedure of MH and ME extracts and yields of obtained fractions.

No.	Fractions	Solvent	Code	Amount (g)	Yield (%)
**MH extract**					
1	1–4	H 100%	D1	2.28	14.84
2	5–13	H 100%	D2	1.13	7.36
3	14–20	HE 10%	D3	3.57	23.24
4	21–47	HE 20%	D4	2.63	17.12
5	48–58	HE 30%	D5	0.52	3.39
6	59–69	HE 40%	D6	0.30	1.95
7	70–81	HE 50%	D7	0.59	3.84
8	82–100	HE 60%	D8	0.81	5.27
9	101–130	HE 70% & HE 80%	D9	0.66	4.30
10	131–152	HE 90% & E 100%	D10	1.34	8.72
11	153–163	EM 50% & M 100%	D11	0.43	2.8
**ME extract**					
12	1–11	H 100% & HE 5%	T1	0.51	5.67
13	12–17	HE 5%	T2	0.14	1.56
14	18–21	HE 5%	T3	0.08	0.89
15	22–26	HE 10%	T4	0.14	1.56
16	27–46	HE 10% & HE 20%	T5	0.74	8.22
17	47–50	HE 20%	T6	0.38	4.22
18	51–63	HE 20%	T7	1.05	11.67
19	64–77	HE 30%	T8	0.99	11.00
20	78–90	HE 30% & HE 40%	T9	0.70	7.78
21	91–101	HE 40%	T10	0.47	5.22
22	102–118	HE 50%	T11	0.60	6.67
23	119–137	HE 60% & HE 70%	T12	0.56	6.22
24	138–156	HE 70%	T13	0.37	4.11
25	157–167	HE 80%	T14	0.20	2.22
26	168–175	HE 90%	T15	0.14	1.56
27	176–181	E 100%	T16	0.15	1.67
28	182–185	MeOH 100%	T17	1.13	12.56

H, n-hexane; HE, n-hexane: ethyl acetate (v/v); EM, ethyl acetate: methanol (v/v); E, ethyl acetate; M, methanol.

**Table 4:** Antioxidant activity of fractions isolated from MH and ME extracts.

Samples	DPPH IC_50_ (mg/mL)	ABTS IC_50_ (mg/mL)	α-Amylase IC_50_ (mg/mL)
**D1**	6.60 ± 0.04 ^k^	12.44 ± 0.56 ^h^	5.72 ± 0.24 ^h^
**D2**	1.64 ± 0.11 ^h,i^	4.54 ± 0.05 ^f^	1.04 ± 0.03 ^i^^,^^j^
**D3**	*ne*	*ne*	0.86 ± 0.01 ^j^
**D4**	*ne*	*ne*	1.47 ± 0.01 ^g,h^
**D5**	*ne*	*ne*	*na*
**D6**	*ne*	*ne*	*na*
**D7**	*ne*	*ne*	*na*
**D8**	*ne*	*ne*	*na*
**D9**	1.41 ± 0.06 ^h,g^	3.54 ± 0.01 ^e^	*na*
**D10**	1.09 ± 0.03 ^f,g^	1.33 ± 0.01 ^c^^,^^d^	*na*
**D11**	1.62 ± 0.02 ^h,i^	1.41 ± 0.03 ^d^	*na*
**T1**	4.36 ± 0.20 ^j^	5.75 ± 0.19 ^g^	2.68 ± 0.05 ^f^
**T2**	1.86 ± 0.23 ^i^	0.31 ± 0.01 ^a,b^	1.29 ± 0.04 ^h,i^
**T3**	0.69 ± 0.01 ^c,d,e^	0.68 ± 0.02 ^ab^	1.03 ± 0.02 ^i,j^
**T4**	0.13 ± 0.01 ^a,b^	0.92 ± 0.02 ^b,c,d^	0.34 ± 0.00 ^k^
**T5**	0.66 ± 0.01 ^c,d,e^	0.54 ± 0.03 ^a,b^	1.78 ± 0.01 ^g^
**T6**	0.45 ± 0.01 ^c,b,d^	0.46 ± 0.02 ^a,b^	10.03 ± 0.05 ^a^
**T7**	0.43 ± 0.00 ^c,b,d^	0.76 ± 0.02 ^b,c,d^	7.86 ± 0.04 ^b^
**T8**	1.82 ± 0.05 ^i^	0.60 ± 0.03 ^a,b^	6.38 ± 0.01 ^c^
**T9**	0.78 ± 0.00 ^c,d,e,f^	0.88 ± 0.01 ^b,c,d^	4.43 ± 0.01 ^e^
**T10**	0.65 ± 0.00 ^c,d,e^	0.89 ± 0.02 ^b,c,d^	*na*
**T11**	0.40 ± 0.00 ^b,c^	0.74 ± 0.01 ^b,c^	*na*
**T12**	0.45 ± 0.00 ^c,b,d^	0.69 ± 0.01 ^a,b,c^	*na*
**T13**	0.47 ± 0.01 ^c,b,d^	0.57 ± 0.05 ^a,b^	*na*
**T14**	0.88 ± 0.01 ^e,f^	0.53 ± 0.01 ^a,b^	*na*
**T15**	0.80 ± 0.01 ^d,e,f^	0.59 ± 0.03 ^a,b^	*na*
**T16**	0.72 ± 0.00 ^c,d,e,f^	0.54 ± 0.02 ^a,b^	*na*
**T17**	0.87 ± 0.00 ^e,f^	0.53 ± 0.01 ^a,b^	*na*
**BHT**	0.02 ± 0.00 ^a^	0.05 ± 0.00 ^a^	-
**Acarbose**	-	-	0.07 ± 0.00 ^k^
**PA**	-	-	1.52 ± 0.03 ^g,h^

The data express means ± standard deviation (SD). Different letters in a column indicate significant differences at *p* < 0.05. MM, total methanolic extract; ME, ethyl acetate extract; MW, water extract; MH, hexane extract; BHT, butylated hydroxytoluence; PA, palmitic acid; “-”, not measured; *ne*, negligible effect; *na*, no activity.

**Table 5:** Major phytoconstituents in the most active fractions T4 and D3.

No.	Identified Compounds	RT (min)	MW (g/mol)	Chemical Classification	Peak Area in Fractions (%)	
					T4	D3
1	Methoxyeugenol	12.03	194	Phenols	-	7.57
2	Myristicin	12.33	192	Benzodioxoles	-	12.82
3	Palmitic acid	17.14	256	Fatty acids	15.11	32.64
4	Seselin	18.19	228	Coumarin	6.71	-
5	13-Tetradece-11-yn-1-ol	18.75	208	Alcohols	-	6.87
6	Linoleic acid	18.77	280	Fatty acids	7.14	-
7	Oleic acid	18.82	282	Fatty acids	4.8	-
8	Braylin	19.57	258	Coumarin	8.48	-
9	Dentatin	21.57	47.32	Coumarin	47.32	-
10	S1	29.54	282	Sesquiterpenes	-	16.69

RT, retention time; MW, molecular weight; “-“, not detected; S1, 2R-acetoxymethyl-1,3,3-trimethyl-4t-(3-methyl-2-buten-1-yl)-1t-cyclohexanol.
